# A Hybrid EMD-Wavelet EEG Feature Extraction Method for the Classification of Students' Interest in the Mathematics Classroom

**DOI:** 10.1155/2021/6617462

**Published:** 2021-01-23

**Authors:** Areej Babiker, Ibrahima Faye

**Affiliations:** ^1^Department of Electronics Engineering, Future University, Khartoum, Sudan; ^2^Centre of Intelligent Signal and Imaging Research & Department of Fundamental and Applied Sciences, Universiti Teknologi PETRONAS, 32610 Seri Iskandar, Malaysia

## Abstract

Situational interest (SI) is one of the promising states that can improve student's learning and increase the acquired knowledge. Electroencephalogram- (EEG-) based detection of SI could assist in understanding SI neuroscientific causes that, as a result, could explain the SI role in student's learning. In this study, 26 participants were selected based on questionnaires to participate in the mathematics classroom experiment. SI and personal interest (PI) questionnaires along with knowledge tests were undertaken to measure student's interest and knowledge levels. A hybrid method combining empirical mode decomposition (EMD) and wavelet transform was developed and employed for feature extraction. The proposed method showed significant difference using the multivariate analysis of variance (MANOVA) test and consistently outperformed other methods in the classification performance using weighted *k*-nearest neighbours (wkNN). The high classification accuracy of 85.7% with the sensitivity of 81.8% and specificity of 90% revealed that brain oscillation patterns of high SI students are somewhat different than students with low or no SI. In addition, the result suggests that the delta rhythm could have a significant effect on cognitive processing.

## 1. Introduction

Learning sciences and mathematics have been an obstacle for many students. Several research studies were carried out to investigate the causes of the low number of students joining these fields. Two out of six suggestions to make mathematics come alive were eliminating mathematics fear and developing interesting teaching strategies to induce positive effect on students' learning [1]. Hence, a variety of researchers proposed the use of situational interest (SI) to get students of different backgrounds to enjoy studying mathematics or science even if they do not have initial or individual interest in the first place [2, 3]. To do so, it is critical to understand the neural mechanisms of interest and curiosity as part of motivational phenomena and their influence on memory and learning as noted by Hidi and Renninger [4]. Therefore, this work explains the effect of SI on student's learning and examines its EEG correlates. The study in [5] investigated the physiological impact of SI but had a relatively small number of subjects and focused on feature extraction with little attention given to EEG power spectra, e.g., delta oscillations. The role of delta EEG rhythm during learning is not clear. The study by Mathewson et al. [6] claimed the possibility of predicting video game learning rate by alpha and delta EEG power. Several studies have reported the increase of delta during tasks that demand attention, cognitive processing, or working memory (see [7] for a review). Yet, the increase of delta is well documented during states like drowsiness, stage 1 sleep, and some brain disorders [8]. Looking at recent approaches in the field of biomedical signal's analysis, there is a trend of combining decomposition methods for feature extraction. This way helps overcoming the weaknesses of each method and enhances their strengths. For example, EMD and wavelet methods were used widely in EEG and ECG research separately or combined depending on the nature of the data and the purpose of the analysis [9, 10]. Because of its features and characteristics, EMD is extensively applied to biomedical signals that are nonlinear and nonstationary time series such as HRV, EMG, and brain signals using EEG. It assumes that every data consist of a number of intrinsic mode functions (IMFs) in which basis oscillation is derived from the data. Similarly, wavelet transform has been used extensively in BCI applications because of its ability to reserve time and frequency information with a wide variety of scale and translation functions. Both methods were successfully used as base for filtering or further feature extraction to achieve high classification accuracy, denoising, and reconstruction of the data with high signal-to-noise ratio. In [11], the authors applied EMD to the seismic signal followed by wavelet ridge to extract the instantaneous spectral properties of a reservoir. They found instantaneous frequency extracted by the wavelet ridge superior to instantaneous frequency extracted by Hilbert transform in revealing geological features. The combination of EMD and wavelet is also used to discriminate between focal and nonfocal EEG signals extracted from epilepsy patients such as the work done by Das and Bhuiyan [12] that used entropy-based features in the combined EMD-DWT space and achieved an accuracy of 89.4%. In [13], wavelet packet transform followed by EMD preceded wavelet packet transform followed by ICA in EEG artifact cleaning. Generally, EMD performance overcame WT in denoising EEG signals as well as preparing feature extraction [14, 15]. Hence, this work is studying the effect of SI in a classroom to examine the SI impact on learning. The experiment was carried out using EEG to acquire the data in order to avoid distraction of lecture flow. EMD and wavelet were used to decompose the EEG data and extract the relevant features for the classification of students' high and low SI. The contribution of this paper can be summarized as follows:

  (1) Identification of EEG correlates corresponding to situational interest in learning in a classroom. This could carry significant information for further understanding of SI phenomena and synchronized EEG recording.
  (2) Discussing and evaluating the potential of employing the hybrid EMD-wavelet approach in extracting the relevant EEG features, especially when certain EEG rhythms or oscillations are required.

## 2. Materials and Methods

### 2.1. Participants

The participants of this experiment were first-year undergraduate and foundation students from Universiti Teknologi PETRONAS (UTP). Students with a history of brain injuries or under any medication that could influence EEG data were excluded. The participants were selected based on a questionnaire regarding joining UTP mathematics club and distributed among all foundation and first-year undergraduate students. In order to select a balanced group of participants with high, low, and moderate interest, the questionnaire was run as a pre-evaluation for the level of personal/individual interest of students. The study ethical approval was obtained from the UTP ethical approval committee, and all participants were familiarized with the experiment and EEG equipment and had given written consent upon their arrival to the experimental room. Twenty-six students participated in this experiment and were remunerated for their time of participation.

### 2.2. Interest Questionnaires and Knowledge Tests

The questionnaires used in this experiment were adopted from published and verified sources. To qualify situational interest, the SI questionnaire designed by Mitchell and Rotgans and Schmidt [2, 16] was adopted. Furthermore, the PI questionnaire in [2] was adopted to qualify individual (personal) interest of the participants. From the situational interest questionnaire result, using the median as the reference, subjects that scored more than 77 out of 100 were considered as high-interest students, while subjects scored less than 69 out of 100 were considered as low-interest students. Subjects scored between 76 and 69 were considered to have moderate situational interest and therefore were not considered for classification tasks.

Because interested students are thought to have better learning compared to noninterested students [17], similar pre- and postknowledge tests were undertaken by students before and after the experiment to evaluate the learning outcome. Both tests consisted of mathematical problems based on the presented lecture.

### 2.3. Stimuli

First-year undergraduate students had a lecture about Laplace transform from the ordinary differential equations (ODE) course that was delivered by the UTP lecturer. The lecture was prepared in an interesting manner by including different examples and changing font and colors. The interestingness of the material was checked in three ways. First, by an expert who viewed the material and approved it, second, by delivering the lecture to different students who did not participate in the experiment and getting their feedback, and the third way of checking the material was by running a 5-Likert-type questionnaire followed by a verbal, nonformal interview at the end of each experimental session. Similar procedure was followed for a lecture on the integration from calculus course for foundation students. Among participants, 83.34% agreed that the lecture was interesting, and all participants agreed that they look forward for similar lectures, which confirmed the interestingness of the stimulation for a majority of the participants.

### 2.4. Experimental Setup and Data Acquisition

**Figure 1:**
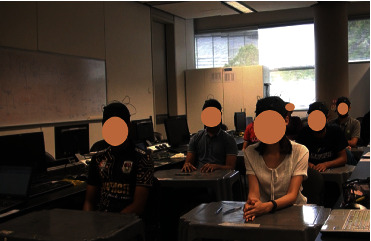
Participants' seating during the experiment.

Due to the limitation of the number of EEG devices, four sessions were run with a maximum number of 10 participants per session. Two cameras in the front and the back of the class were settled for video recording throughout the experiment. These video data were used later to confirm the self-reported interest result when needed. Upon the arrival of participants and signing of the consent forms, preknowledge test and PI questionnaire were undertaken. This was to ensure low to no knowledge about the presented topic and to confirm the level of individual interest. Then, participants wore Enobio EEG caps simultaneously, which had 8 channels each, dry sensors, and a sampling frequency of 500 Hz as shown in Figure 1, with the researcher's assistance and were ought to speak to researchers if they felt discomfort and had the option to leave the experiment at any time. The EEG channels included Fp1, Fp2, F3, F4, T7, T8, O1, and O2 according to the 10–20 international system, and the reference electrodes of common mode sense (CMS) and driven right leg (DRL) in which both were placed in the right mastoid were employed [16].

**Figure 2:**
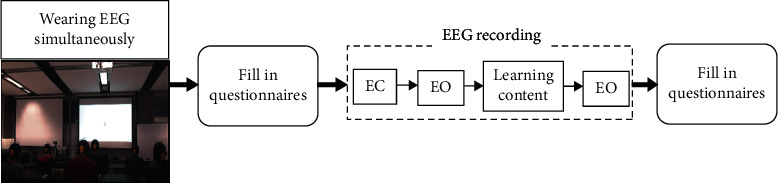
Experiment block diagram. EC: eyes-closed; EO: eyes-opened; learning content: Laplace transform/integration.

The baseline data of 4 min eyes-opened and 4 min eyes-closed were acquired followed by about 22 min of Laplace transform lecture or integration lecture. Then, another baseline of 4 min eyes-opened was recorded. The presentations were delivered through a projector to a projector screen. After the EEG recording, SI questionnaire and postknowledge test were undertaken. This was followed by a presentation questionnaire to evaluate the interestingness of the topic.  Figure 2 shows the experiment block diagram.

### 2.5. Data Preprocessing

Two subjects' data were removed because of technical errors that caused either faulty EEG data embedded with DC components or corrupted EEG file and, therefore, were not appropriate for analysis. The recorded videos were used to mark the exact time of starting and ending the lecture and observe the behaviour of students during the experiment.

In the obtained EEG data, drift was corrected, and a notch filter of 50 Hz was applied to remove line noise. The data were then filtered using the FIR filter with a low frequency of 0.5 Hz and high frequency of 47 Hz. After that, the region of interest (ROI) was extracted as follows: for high SI, the moments where subjects expressed high interest in the content were selected. For low SI, the moments of no interest in the presented content were selected.

The moments in which subjects were not expressing high or low interest or the moments in which the subject's face was not clear or not shown were excluded from the analysis. The number of segments derived varied from one subject to another because of individual interest differences. Therefore, the least length among subjects was set as the standard length in order to have equal length of data from all the subjects to ensure unbiased analysis. This length was 2 min and 34 s and was extracted from all the subjects.

### 2.6. Feature Extraction Methods

#### 2.6.1. Power Spectral Density

To obtain the power spectral density, the raw EEG data were filtered according to respective frequencies, gamma, beta, alpha, theta, and delta. The power was calculated using the periodogram method by Welch [18] through Hanning window function. First, the EEG data were segmented into eight segments with 50% overlap. Then, power spectral density (PSD) was calculated for each one of these segments. After that, the average PSD was calculated for all the segments to obtain the absolute power for each wave band.

The calculated EEG power of each wave was then averaged across brain regions, i.e., frontal (Fp1, Fp2, F3, and F4), temporal (T3 and T4), and occipital (O1 and O2) to graph the differences in brain regions between high and low SI students. This procedure was performed for the baseline condition (4 min eyes-opened) and lecture condition (Laplace/integration). After that, the percentage of change was obtained by subtracting the PSD of the lecture condition from the PSD of the baseline and dividing the result by the PSD of the baseline. This step is necessary to account for subjective variability.

#### 2.6.2. Empirical Mode Decomposition

EMD is proposed to decompose the EEG signals: 1) to reduce the signal noise and (2) to increase the number of features and improve the analysis result by separating the main signal frequency into subband frequencies. The resulted oscillations are independent of each other and might be linear or nonlinear with the same number of extrema and zero-crossings [19, 20]. IMFs are nearly periodic oscillations with zero mean. Hence, each IMF follows the following: (1) the number of extrema and zero-crossings must equal or differ by no more than one in the data; (2) the mean value of local maxima and local minima envelopes is equal to zero. These IMF values can be found by generating the upper and lower envelops of the EEG signal *X*(*t*) by finding all the local extrema and interpolating them with a cubic spline line. The mean of the upper and lower envelops *m*_1_(*t*) is used to produce the first component, *h*_1_(*t*):

(1) $$\begin{array}{c} X\left(t\right)-m_{1}\left(t\right)=h_{1}\left(t\right). \end{array}$$

New *h*_1_(*t*) is subtracted from the mean until it complies with the two conditions described above. Then, the first IMF is produced as IMF1 = *c*_1_(*t*). This *c*_1_(*t*) is subtracted from *X*(*t*) yielding a residue *r*_1_(*t*). The residue now becomes the new signal *X*(*t*), and the procedure is repeated until residue signal *r*_1_(*t*) becomes monotonic or no more IMFs can be derived from it. Then, the sifting process stops to finally obtain

(2) $$\begin{array}{c} X\left(t\right)=\sum_{j=1}^{n}c_{j}\left(t\right)+r_{n}\left(t\right). \end{array}$$

The decomposition is achieved by having *n* empirical modes and one residue *r*_*n*_(*t*) that reflects a constant value or the average trend of *X*(*t*).

#### 2.6.3. Discrete Wavelet Transform

Discrete wavelet transform is a transform that decomposes a signal into its low- and high-frequency components using a specific subset of frequency and translation values determined by the type of data and purpose of decomposition. By determining the wavelet mother, the EEG signal is decomposed up to the predetermined decomposition level using equations (3) and (4) by designed low- and high-pass filters producing detail *D*_*j*_ and approximation *A*_*j*_ coefficients for each level, where *j* represents the decomposed level. The approximation *Aj* is then used for further decomposition, and the maximum decomposition level depends on the principal frequency of the signal.

(3) $$\begin{array}{c} \phi_{j,k}\left(n\right)=2^{\left(j/2\right)}h\left(2^{j}n-k\right), \end{array}$$

(4) $$\begin{array}{c} \psi_{j,k}\left(n\right)=2^{\left(j/2\right)}g\left(2^{j}n-k\right), \end{array}$$

where *n* = 0, 1, 2, .., *M* ‒ 1, *j* = 0, 1, 2,…, *J* − 1 with *J* = number of decomposition levels, *k* = 0, 1, 2,…,  2^*j*^ − 1, and *M* is the length of the EEG signal *x*(*n*). Denoting the high-pass filter as *g* (*n*) and low-pass filter as *h* (*n*), the dilation function and the wavelet function can be written as follows [21]:

(5) $$\begin{array}{c} A_{j}=\frac{1}{\sqrt{M}}\sum_{n}x\left(n\right).\phi_{j,k}\left(n\right),\\ D_{j}=\frac{1}{\sqrt{M}}\sum_{n}x\left(n\right).\psi_{j,k}\left(n\right), \end{array}$$

where *x*(*n*) represents the EEG signal with a length of *M* and *φ*_*j*,*k*_(*n*) and *ψ*_*j*,*k*_(*n*) represent the dilation and mother function of the wavelet, respectively.

#### 2.6.4. Proposed EMD-Wavelet Energy

Using EMD, the preprocessed EEG data were decomposed into several IMFs with a residue. The signal is decomposed by direct extraction of the local energy associated with signals' time scales. Each IMF contains single-frequency or limited frequency bands that allow better representation of the EEG signal. Furthermore, the data contain some important information that could be regarded as artifact, but it is important for the classification, and the use of EMD improves the signal-to-noise ratio while keeping this information. For example, the quantity and quality of eye blinks and body movement could be related to student's interest, and the removal of this information could cause loss of significant classification features. With EMD, the whole signal is decomposed into the IMFs that can be later used efficiently to construct back the signal with some noise removed [18]. After extracting the IMFs, DWT was applied for each IMF to obtain the percentage of energy (relative energy) corresponding to the approximation and use it as a feature. The approximation of the DWT is used in several studies to construct a noise-free signal/image because it reserves its properties.

By checking the result of several wavelets, Daubechies with 5 decomposition levels was found to be appropriate for obtaining reliable feature vectors from the EEG signal [22]. Daubechies wavelet is the best choice among other mother wavelets when reserving signal energy is required; besides, it has strict vanishing moment. In addition, five levels of decomposition correspond to the basic EEG rhythms: delta, theta, alpha, beta, and gamma, which offer proper selection of the required rhythm or wave band.

The corresponding detail and approximation coefficients of the EEG signal using db4 with 5 decomposition levels are shown in Table 1. The frequency bands of each decomposition level comprised in the range [*f*_*m*_/2: *f*_*m*_] such that *f*_*m*_ = *fs*/2^*j*+1^, where *f*_*s*_ is the sampling rate and *j* is the level of decomposition [23].

After that, the approximation was used to calculate the wavelet energy through the following equation [24]:

(6) $$\begin{array}{c} EA_{i}=\sum_{j=1}^{N}{\left|A_{ij}\right|}^{2},\; \end{array}$$

where *i* = 1, 2, .., *l* is the number of coefficients by level which are kept in vector *l* and *N* is the signal's length.

The percentage of energy of the approximation coefficients at the coarsest scale for each decomposed IMF was then arranged in a feature vector for classification. This was performed for each EEG channel producing a total of 64 features per segment.

The main advantage of this method is to precisely extract the energy of the signal corresponding to the EEG delta rhythm. Also, the method does not require removal of artifacts due to the 2-stage filtering using EMD and DWT which makes it favourable for online and classroom analysis.

### 2.7. Classification

*k*-nearest neighbours is a discriminant classifier that classifies an unseen point from the testing set based on the dominant class of its nearest neighbours. By manipulating the *k* values, kNN can produce a nonlinear decision boundary.

kNN uses nonparametric density estimation and, therefore, better fits the actual densities encountered in practice. Euclidean distance in equation (7) is used to determine the *k*-nearest neighbours of the unseen instance:

(7) $$\begin{array}{c} \text{dist}\left(x_{i},y_{i}\right)=\sqrt{\overset{n}{\underset{i=1}{\sum }}{\left(x_{i}-y_{i}\right)}^{2}}, \end{array}$$

where *i* *=* 1, 2, 3,…, *n* is the number of points in a vector.

kNN has different types: one of them is weighted kNN (wkNN). The idea of weighted kNN is that each observation from the training set that is close to a new observation should get a high weight in the decision, while the observation that is far away should get lower weight in the decision. The main difference between kNN and wkNN is that kNN is influenced only by the *k* neighbours close to the observation to make the decision regardless of the individual similarities, while wkNN gives weight to each observation based on how close they are to the training observations, and the higher weights make the decision. Thus, wkNN overcomes the limitation of kNN and improves the classification accuracy. To determine the weight, the following equation is simply used:

(8) $$\begin{array}{c} \text{weight}\left(x_{i},y_{i}\right)=\frac{1}{{\left(\text{dist}\left(x_{i},y_{i}\right)\right)}^{2}}, \end{array}$$

where the distance between the two points *x*_*i*_ and *y*_*i*_ is calculated using equation (7), and therefore, the number of *k* can now be automatically selected.

## 3. Results and Discussion

### 3.1. Behavioural Analysis

Examining behavioural data, which are postknowledge tests and SI questionnaires, showed a positive relationship between the high score in the SI questionnaire and the high postknowledge test score.

The correlation value between postknowledge test and situational interest in exp. 1 and exp. 2 was *r* = 0.603 and *r* = 0.561, respectively, with *p* < 0.05 indicating a moderate positive relationship. That is, the increase in situational interest has a positive effect on postknowledge test. This is in line with the previous subjective research studies that showed a positive correlation between situational interest and academic achievement [16, 17].

### 3.2. Power Spectral Analysis

The result of the PSD was averaged across brain lobes to identify the specific brain region that represents the significant change between high and low SI subjects.  Table 2 presents the significant difference that occurred in two brain lobes (frontal and occipital) and in the theta rhythm in the occipital lobe.

**Figure 3:**
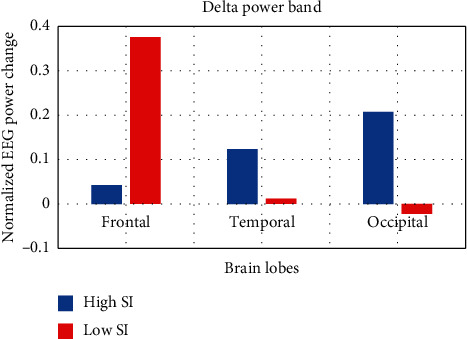
PSD change of delta in frontal, temporal, and occipital lobes between high SI and low SI students.

Figure 3 shows an increase of the delta wave in the frontal lobe in the lecture condition compared to the baseline condition for both groups. However, this increase was significantly higher for the low SI group in the frontal lobe accompanied by a significant decrease in the occipital lobe. Since processing of visual content such as colors and shapes in the presentation slides is performed mostly in the occipital lobe, it is rational to suggest that high SI students were more focusing and paying attention to the presented materials compared to low SI students of whom some of them were sleepy or fell asleep as recorded by the cameras during the lecture. Moreover, the concomitant change of delta activation in frontal and occipital lobes could indicate attention to the presented material as described in [25]. Increase of delta of about 1 Hz in parietal and temporal lobes was reported during Go/No-Go tasks that require cognitive processing [7].

The results obtained thus far show the potential for using delta rhythm to extract features related to situational interest. To extract the delta rhythm, an efficient method has to be used to obtain good size of data. Extracting delta rhythm using EMD is possible but will result in low amount of data because only the lower one or two IMFs can carry the delta band (<4 Hz). Similarly, for DWT, the last approximation component can be regarded as delta as in Table 1. However, combining EMD and wavelet as described earlier in the proposed EMD-wavelet energy section offers good quality and quantity of EEG data in the delta rhythm. This is because EMD decomposes the EEG signal into its empirical modes, and then from each mode, the low-frequency component represented by the wavelet approximation is extracted. In another word, employing EMD followed by wavelet decomposes the EEG into its modes starting from high-frequency components to low-frequency components and then extracting the low-frequency components from each mode. This way, components such as IMF 1 and IMF 2 that contain high frequency can be used because it will be followed by wavelet decomposition to obtain the approximation which contains the low-frequency component of the signal.

### 3.3. Statistical Analysis

In order to assess the discrimination ability of the features extracted using the proposed method, it was compared to another two methods which are the energy of EMD using all the 8 IMFs (EMD-energy) and the energy of all the details and approximation of DWT (DWT-energy). EMD-energy produced 8 features per channel, i.e., 8 features *×* 8 channels = 64 features, while DWT-energy produced 6 features *×* 8 channels = 48 features. To determine the significance of the results obtained, two-way multivariate analysis of variance (MANOVA) was applied. The two independent variables were lecture type (Laplace/integration) and situational interest (low/high), while the dependent variables were the extracted features.  Table 3 summarizes the number of features of each method and the corresponding MANOVA result per data segment for the three methods (including the proposed method).

Table 3 shows significant difference of the extracted features between high and low SI students only when using the proposed method with *p*=0.008. EMD-energy had less *p* value compared to DWT-energy indicating better discrimination ability. This is because the EMD method has approximately 4 out of the 8 decomposed IMFs falling in the delta range, while DWT, as shown in Table 1, has only one decomposed approximation falling in the delta range which implies the inclusion of nonsignificant features that belong to the other EEG rhythms (alpha, beta, and gamma).

Hence, the proposed method achieved both objectives which are selecting the rhythms related to SI and increasing the number of these relevant features.

### 3.4. Classification

The selected significant features are now ready for classification. The features extracted by the two aforementioned methods along with the proposed method were classified using linear SVM and weighted kNN. The result of the classification is shown in Table 4.

The results in Table 4 show the superiority of the proposed method in classifying students' high and low SI interest. The other two methods show either low sensitivity or low specificity indicating high misclassification between the two groups. The highest accuracy of 85% was achieved using weighted kNN, while the result of linear SVM was quite poor (<67%) which suggests the superiority of weighted kNN in this case.

There are some limitations to the current study. The two experiments were carried out in different classrooms which are slightly different in terms of lightening and available space. Future studies are encouraged to fix the experiment room and perhaps repeat the experiment with the same participants. Also, to address the phenomenon of interest, it is advised to use an individual experiment along with the classroom to account for possible EEG group-based synchronization or coherence impact; see [26, 27] for a review.

## 4. Conclusion

This research is conducted to address the changes between high and low SI students using EEG. The research proposed a novel approach for extracting the features from raw EEG data. The results supported the claim that high SI students show some different brain activities compared to low SI students. The extracted features relevant to the delta rhythm achieved high classification accuracy that reached 85.7% using EMD-wavelet energy features fed into the weighted kNN classifier. Behavioural analysis revealed a positive relationship between students' postknowledge test score and high SI suggesting that students with high SI are most likely to have high test scores and, as a result, more knowledge compared to low SI students.

## 5. Disclosureback

The funders had no role in the study design, data collection and analysis, decision to publish, or preparation of the manuscript.

## Figures and Tables

**Table 1:** The corresponding frequency obtained using db4 for 5 decomposition levels.

Level	Wavelet coefficients	Frequency (Hz)	EEG rhythms	EEG frequency (Hz)
1	*D*_*j*=0_	62.5–125	—	—
2	*D*_*j*=1_	31.25–62.5	Gamma	>30
3	*D*_*j*=2_	15.62–31.25	Beta	13–30
4	*D*_*j*=3_	7.81–15.62	Alpha	8–12
5	*D*_*j*=4_	3.91–7.81	Theta	5–7
5	*A*_*j*=4_	1.91–3.91	Delta	<4

**Table 2:** The result of the *t*-test for the PSD between high and low SI groups.

Channel	*p* value				
	Delta	Theta	Alpha	Beta	Gamma
Fp1	0.054	0.074	0.144	0.152	0.159
Fp2	0.022	0.162	0.390	0.121	0.638
F3	0.378	0.651	0.387	0.307	0.526
F4	0.140	0.180	0.158	0.210	0.424
T7	0.065	0.172	0.316	0.277	0.227
T8	0.631	0.519	0.696	0.754	0.973
O1	0.118	0.328	0.952	0.594	0.332
O2	0.029	0.011	0.367	0.363	0.235

**Table 3:** MANOVA result of the features of high and low SI students.

Method	Independent variables	MANOVA
EMD-energy	Interest	*F* (16, 1) = 9.024, *p*=0.256, Wilks' ∧ = 0.007
	Lecture	*F* (16, 1) = 0.229, *p*=0.947, Wilks' ∧ = 0.214; interaction *p*=0.097
DWT-energy	Interest	*F* (16, 1) = 2.063, *p*=0.504, Wilks' ∧ = 0.029
	Lecture	*F* (16, 1) = 6.376 *p*=0.303, Wilks' ∧ = 0.010; interaction *p*=0.276
Proposed method	Interest	*F* (10, 7) = 7.275, *p*=0.008, Wilks' ∧ = 0.088
	Lecture	*F* (10, 7) = 12.818, *p*=0.001, Wilks' ∧ = 0.052; interaction *p*=0.506

**Table 4:** Comparison of the classification accuracy using linear SVM and weighted kNN.

Method	Linear SVM			Weighted kNN		
	Acc. (%)	Sens. (%)	Spec. (%)	Acc. (%)	Sens. (%)	Spec. (%)
EMD-energy	66.7	90.9	40	57.1	81.8	30
DWT-energy	57.1	54.5	60	66.7	54.5	80
Proposed method	61.9	54.5	70	85.7	81.8	90

Acc. = accuracy; Sens. = sensitivity; Spec. = specificity.

## Data Availability

The datasets generated during the current study are available from the corresponding author upon reasonable request.
